# Addressing the challenge of platinum-resistant ovarian cancer: the role of mirvetuximab soravtansine

**DOI:** 10.1097/MS9.0000000000002759

**Published:** 2025-01-09

**Authors:** Ayesha Shaukat, Laiba Shakeel, Afsheen Khan, Hamza Irfan, Aymar Akilimali

**Affiliations:** a Department of Internal Medicine, Dow University of Health Sciences, Karachi, Pakistan; b Department of Internal Medicine, Shaikh Khalifa Bin Zayed Al Nahyan Medical and Dental College, Lahore, Pakistan; c Department of Research, Medical Research circle, Goma, Democratic Republic of the Congo

## Introduction

Ovarian cancer, commonly known as the “silent killer” because it often presents without noticeable symptoms, poses a major health risk for women globally. It is the sixth most prevalent cancer in developed nations^[1]^. The prevalence and incidence of ovarian cancer vary by region, with developed countries showing higher rates. According to recent statistics, approximately 313 959 cases are reported annually, and 207 252 succumbed to death, highlighting its severe mortality rate^[2]^. Ovarian cancer prevalence varies significantly worldwide, with the highest rates in Western Europe and North America, moderate levels in Southern and Eastern Europe as well as South America, and the lowest rates observed in the Middle East and Asia^[1]^.

In the United States, approximately 19 680 new cases of ovarian cancer are expected, and 12 740 deaths from the disease are projected in 2024 by the American Cancer Society. Ovarian cancer constitutes 2.5% of all cancers affecting women, ranking as the eleventh most prevalent malignancy in this demographic^[3]^. According to the Centers for Disease Control and Prevention (CDC), it ranks as the second most prevalent cancer in the United States^[4]^. Additionally, it ranks as the fifth leading cause of cancer-related deaths among women in the United States and is regarded as the deadliest of all gynecologic cancers^[5]^.

One of the primary challenges associated with ovarian cancer is the frequent late-stage diagnosis. In most developed countries, the median age at diagnosis is approximately 63 years, and ovarian cancer is more prevalent in older women than in younger women^[1]^. Early-stage ovarian cancer typically manifests with vague symptoms such as urinary urgency, bloating, and pelvic pain, which are often mistaken for benign conditions. This misdiagnosis contributes to a poorer prognosis and lower survival rates^[6]^.

Ovarian cancer is associated with certain risk factors that can complicate its early detection. Factors such as age, genetic predisposition, such as BRCA gene mutations, both 1 and 2, family history of ovarian or breast cancer, and reproductive history significantly influence the likelihood of developing the disease^[1]^. It is a multifaceted disease that can arise at any age and can originate from different cell types within the ovary, such as oocytes, granulosa cells, theca interstitial cells, and the surface epithelium^[1]^.

## Classification of ovarian cancer

The most recent classification by the WHO recognizes five primary histological types of ovarian cancer: clear cell carcinoma, endometrioid carcinoma, mucinous carcinoma, low-grade serous carcinoma (LGSC), and high-grade serous carcinoma (HGSC)^[1]^. Among the various subtypes of ovarian cancer, epithelial ovarian cancer is the most prevalent, representing approximately 90% of cases. This subtype arises from the cells lining the outer surface of the ovaries. Other, less common subtypes include germ cell tumors, which develop from the egg-producing cells, and stromal tumors, which originate from the supportive tissue cells that maintain the ovary’s structure and produce female hormones^[1]^.

Significant advancements in understanding tumor behavior and identifying potential treatment options have been made, particularly in HGSC, through the molecular classification of ovarian cancer. HGSC is mainly categorized into four subtypes according to gene expression profiles and fundamental biological features. The immunoreactive subtype is characterized by high immune cell infiltration and immune-related gene expression, suggesting potential sensitivity to immunotherapy. The proliferative subtype exhibits high levels of cell proliferation markers, often associated with rapid tumor growth and worse overall survival (OS). In contrast, the differentiated subtype represents a more differentiated tumor profile with lower proliferation levels, generally correlating with better prognosis. The mesenchymal subtype shows features of epithelial-to-mesenchymal transition, often linked to a more aggressive phenotype and varied therapeutic responses^[7]^.

Beyond HGSC, other ovarian cancer types also possess distinct molecular features. For instance, LGSC is often driven by mutations in BRAF, KRAS, and ERBB2, presenting a less aggressive clinical course compared to HGSC^[8]^. Clear cell carcinoma frequently harbors mutations in ARID1A and PIK3CA, often displaying unique metabolic profiles and resistance to standard chemotherapy^[9]^. Endometrioid carcinoma may share mutations with endometrial cancer, such as PTEN and mismatch repair genes^[10]^.

Our study examines mirvetuximab soravtansine-gynx (MIRV), an FDA-approved drug, evaluating its properties, dosage, mechanism of action, and potential advantages for treating ovarian cancer. It seeks to offer essential insights into this therapeutic option, assisting individuals in understanding the possible benefits of MIRV in addressing the rising rates of ovarian cancer.

## Pathogenesis of ovarian cancer

Several theories have been proposed to explain the pathogenesis of ovarian cancer^[11]^. These include the fallopian tube, incessant ovulation, and two-pathway theories.

### Incessant ovulation theory

According to incessant ovulation theory ovarian cancers arise from the ovarian surface epithelium, which undergoes repetitive trauma and DNA damage during ovulation. This damage leads to the formation of cortical inclusion cysts that can transform into cancer cells due to hormonal exposure. However, this theory fails to account for the pathogenesis of different histological types of ovarian cancer or the variations in prognosis. Additionally, it contradicts the increased risk of ovarian cancer observed in patients with polycystic ovary syndrome, despite their fewer ovulation cycles^[11]^.

### Fallopian tube theory

This theory suggests that ovarian cancer frequently develops from early-stage lesions in the fallopian tubes, particularly in women carrying BRCA1/2 mutations. Studies have found epithelial dysplasia in the Fallopian tubes resembling HGSC, known as tubal intraepithelial carcinoma (TIC). TP53 gene mutations are present in TIC, similar to those in serous ovarian cancer. The fimbria region of the Fallopian tube, rich in blood vessels, is a common site for TIC and is believed to facilitate metastasis to the ovaries^[11]^.

### Two-pathway theory

**Figure 1.**
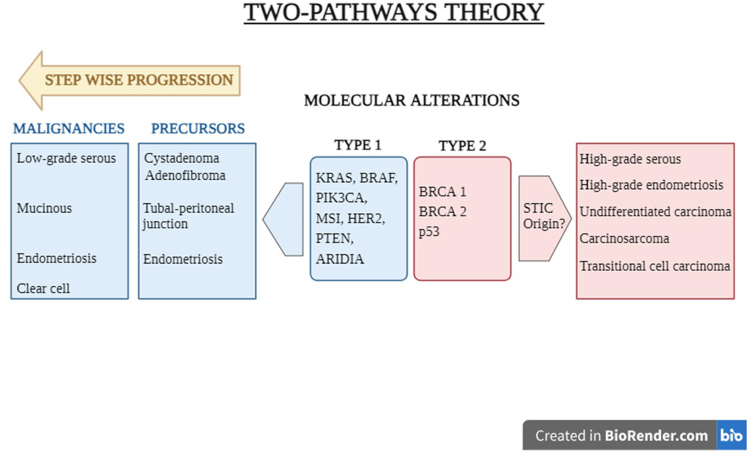
The two-pathways theory of ovarian cancer.

The two-pathway theory was proposed in 2004, which categorizes ovarian cancer into type I and type II based on histological, genetic, and clinical characteristics (Fig. 1). Type I includes low-grade serous, mucinous, endometrioid, clear cell, and transitional histologies originating from the ovary, growing slowly, and being genetically stable, with common KRAS and BRAF mutations. Type II comprises undifferentiated, high-grade serous, and carcinosarcoma histologies, often originating from the fallopian tube, growing aggressively, and being genetically unstable, with frequent TP53 mutations and BRCA1/2 gene associations. This theory explains the pathogenesis of ovarian cancer more comprehensively than others but still lacks an understanding of non-ovarian origins^[11]^.

## Detection and diagnosis of ovarian cancer

The symptoms associated with ovarian cancer lack specificity, posing a challenge in early detection, as they can mimic those of other medical conditions. Typically, these symptoms manifest prominently in the late stages of the disease (stage III or stage IV)^[12]^. Currently, screening methods for ovarian cancer encompass transvaginal ultrasound (TVU), physical examination, and serum biomarkers estimation, chiefly CA125^[13]^. However, the limited sensitivity of CA125 in detecting early-stage disease necessitates exploring alternative biomarkers such as cancer antigen 19-9 (CA19-9) and human epididymis protein 4, which demonstrate potential for identifying specific subtypes^[14]^.

Advancements in imaging modalities, including Doppler ultrasound to enhance TVU accuracy, high-resolution imaging via MRI, and PET/CT scans amalgamating structural and functional data, offer improved diagnostic capabilities. Emerging hysteroscopy techniques employing Falloposcope devices aim to procure cytological samples from the fallopian tubes, presenting a promising avenue for early screening and diagnosis^[15]^. Concurrently, the evolving landscape of biomarkers, encompassing miRNA, the cancer microbiome, autoantibodies, extracellular vesicles, and metabolomics, is reshaping ovarian cancer diagnostic strategies. Additionally, advancements in imaging technologies hold potential for augmenting precision and sensitivity in detecting minimal disease volumes.

## Current treatment overview for ovarian cancer

The management of ovarian cancer typically involves a two-pronged approach, which involves initial surgery followed by chemotherapy. The chemotherapy regimen is predominantly platinum-based and is administered intravenously, often in conjunction with taxane drugs. This treatment is delivered in six cycles, each spaced three weeks apart^[16]^. However, in cases involving less aggressive tumors (G1/G2) or early-stage cancers (IA/IB), chemotherapy may be deemed unnecessary. As the disease progresses, achieving complete tumor removal becomes increasingly difficult, particularly when tumors invade critical areas such as the liver or small bowel mesentery^[16]^. In such advanced cases, the first line of action often involves chemotherapy to shrink the tumor before considering surgical options. If a positive response is observed after three cycles, surgery may be feasible, followed by additional chemotherapy^[16]^. Despite the potential for a complete response to initial treatment, the risk of recurrence remains a significant concern for many patients.

The interval between treatments, known as the treatment-free interval, along with the tumor’s response to platinum-based drugs, plays a vital role in determining prognosis^[16]^. The efficacy of platinum chemotherapy largely depends on the speed of tumor relapse, which serves as an indicator of its sensitivity to these agents. This sensitivity is crucial for guiding subsequent treatment decisions. For tumors that remain responsive to platinum, combining it with other agents such as gemcitabine, paclitaxel, or pegylated liposomal doxorubicin (PLD) has been found beneficial for managing both highly and partially responsive malignancies^[16]^. In cases where platinum is no longer effective, an alternative approach may involve combining PLD with trabectedin for partially sensitive recurrences. Unfortunately, patients who exhibit resistance or refractoriness to platinum treatments tend to have a poorer prognosis^[16]^.

In cases where chemotherapy is combined with bevacizumab, there is evidence of significantly extended progression-free survival (PFS) for patients maintaining good performance status. Furthermore, in selected instances of recurrent ovarian cancer, surgical intervention may still offer an opportunity for radical treatment, complete remission, and potentially a year-long respite from the disease post-initial therapy^[16]^.

### Mechanisms of resistance

Platinum-based drugs achieve their anticancer activity by binding covalently to DNA, which interferes with its replication and promotes cell death by generating cross-links. However, resistance to these treatments can arise from various factors, including immune responses, epigenetic modifications, genetic alterations, and environmental influences^[17]^. The effectiveness of platinum-based chemotherapy is closely tied to a cell’s ability to recognize and repair drug-induced DNA damage, a process regulated by the DNA damage response mechanism^[18]^. Dysregulation of this mechanism can lead to either increased susceptibility to the drugs or the development of resistance. In platinum-resistant cancers, overexpression of DNA repair proteins such as BRCA 1/2, MSH1, MSH2, ERCC proteins, RAD51, and Fanconi anemia complementation group D2 is often observed^[17]^.

Moreover, alterations in drug transport channels can disrupt the influx and efflux of platinum salts within cancer cells, resulting in diminished concentrations of cisplatin and leading to unrepaired DNA lesions^[19]^. While platinum-based chemotherapy continues to serve as a cornerstone of cancer treatment, its limitations underscore the necessity for more targeted interventions. By directly addressing the molecular pathways that drive cancer growth and survival, targeted therapies have the potential to minimize damage to healthy tissues, overcome drug resistance, and improve long-term patient outcomes.

## Mirvetuximab soravtansine-gynx as drug therapy

**Figure 2.**
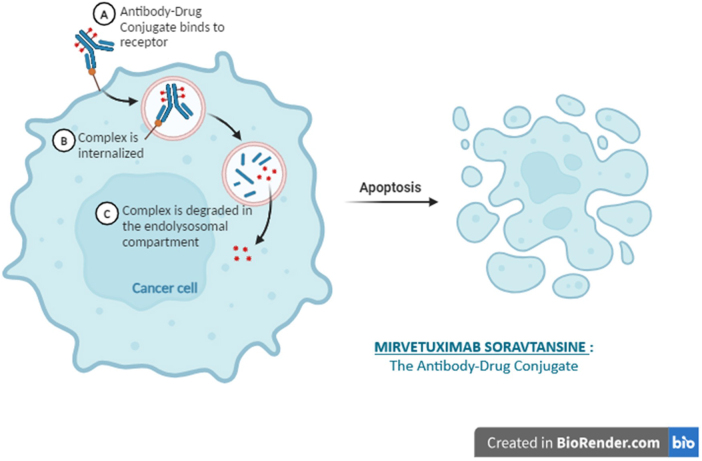
The mechanism of action of MIRV.

Recently, MIRV has been approved for adult patients with Folate receptor (FR) α-positive, PROC, primary peritoneal and tubular cancer by the Food and Drug Administration (FDA), for people who have undergone one to three prior treatment courses^[20]^. This antibody–drug conjugate (ADC) includes a chimeric anti-FRα monoclonal antibody (IgG1 subtype), a chemically synthesized microtubule inhibitor DM4, and a chemically synthesized cleavable linker, sulfo-SPDB^[10]^. MIRV specifically targets FRα on the surface of cells, leading to the uptake of the receptor–ADC complex and its subsequent degradation within lysosomes^[21]^. This mechanism releases the DM4 payload, which triggers cell cycle arrest and ultimately results in cell death (Fig. 2). In vivo research has demonstrated anti-tumor effectiveness in mouse xenograft models of FRα-positive ovarian cancer^[21]^.

MIRV’s mechanism of action combines targeted delivery with cytotoxic effects, positioning it as a useful treatment option for patients with platinum-resistant ovarian cancer (PROC). Its structure includes a humanized monoclonal antibody that specifically attaches to FRα, a cleavable linker, and a powerful cytotoxic component called DM4, a type of maytansinoid^[22,23]^. Targeting FRα is crucial because this receptor is often overexpressed in ovarian tumors, allowing for the precise delivery of the cytotoxic drug directly to cancer cells while reducing harm to normal tissues^[24,25]^. This targeted approach minimizes systemic toxicity, which is a common drawback of conventional chemotherapy^[22,23]^. When MIRV attaches to FRα on tumor cell surfaces, it undergoes uptake through receptor-mediated endocytosis. After entering the cell, the cleavable linker becomes activated, releasing the DM4 payload. DM4 disrupts microtubule dynamics, leading to cell cycle arrest and ultimately triggering apoptosis in the cancer cell^[23,24]^. This mechanism is particularly effective in tumors that express high levels of FRα, as it ensures that the cytotoxic effects are concentrated where they are most needed^[22,23]^.

Besides its direct cytotoxic effects, MIRV has been demonstrated to activate immune responses. Its binding to FRα can trigger antibody-dependent cellular cytotoxicity and phagocytosis, boosting the immune system’s capacity to identify and eliminate cancer cells^[22,26]^. This dual mechanism of action – direct cytotoxicity combined with immune activation – provides a comprehensive approach to treating ovarian cancer.

## Evidence from the clinical trials

The FDA evaluated the pharmacology of MIRV by analyzing detailed data from five studies: Study 0401 (NCT01609556), 0416 (NCT04209855), Study 0417 (NCT04296890), Study 0403 (NCT02631876), Study and Study 0402 (NCT02606305) (Table 1)^[22]^. In 2017, Study 0401 showed promising results for MIRV in FRα-positive PROC. However, Study 0403 did not find a considerable benefit in FRα-positive tumors. As a result, MIRV was then tested only in the FRα-high population in Study 0417, using a different folate receptor test and achieved positive results^[22]^.

**Table 1:** **Clinical trials testing MIRV in platinum-resistant ovarian cancer^[22]^**.

Clinical Trial ID	NCT01609556	NCT02631876	NCT04296890	NCT02606305	NCT04209855
Phase	Phase I	Phase III	Phase II	Phase Ib/II	Phase III
Patients	46 pts receiving mirvetuximab	366 pts (243 receiving mirvetuximab)	106 pts receiving mirvetuximab	94 pts receiving mirvetuximab plus bevacizumab	227 pts receiving mirvetuximab
≥3 previous lines of therapy	>50%^a^	86 (34%)	54 (51%)	49 (52%)	105 (46%)
Previous bevacizumab exposure	NR	121 (48.8%)	106 (100%)	55 (59%)	138 (61%)
Previous PARPi exposure	NR	44 (17.7%)	51 (48%)	25 (27%)	124 (55%)
ORR	26.1%	22%	30.2%^b^	44%	42%^c^
Complete response	1%	NR	5 (4.8%)	5 (5%)	12 (5%)^c^
Partial response	11%	NR	29 (27.6%)	36 (38%)	84 (37%)^c^
Stable disease	28%	NR	48 (45.7%)	44 (47%)	86 (38%)^c^
Progressive disease	4%	NR	20 (19.0%)	8 (9%)	31 (14%)^c^
Unknown	2%	NR	3 (2.9%)	1 (1%)	14 (6%)^c^
mDOR	19.1 weeks	5.7 months	NR	9.7 months	NR
PFS (months)	4.8	4.1	5.5	8.2	5.9^d^
OS (months)	NR	16.4	13.8	NR	16.46^d^
Patients with FRα high	23 (50%)	147 (60.4%)	105 (99%)	44 (46.8%)	227 (100%)
ORR in FRα high subset	26.1%	24%	30.2%	48%	36%^d^
PFS in FRα high subset (months)	NR	4.8	5.5	9.7	5.9^d^
OS in FRα high subset (months)	NR	NR	13.8	NR	16.46^d^

Key:

^a^ > 50% of patients received ≥3 previous lines of therapy.

^b^ Includes a subset of patients.

^c^ Data from interim analysis.

^d^ Revised data.

FRα, folate receptor alpha; mDOR, median duration of response; NR, not reported; ORR, objective response rate; OS, overall survival; PARPi, poly (ADP-ribose) polymerase inhibitors; PFS, progression-free survival; pts, patients.

### Drug efficacy

The approval of MIRV was largely contingent upon the findings from a multicenter, randomized trial that included 453 patients with PROC, primary peritoneal or tubular cancer. Participants could have received up to three previous instances of systemic therapy and were chosen based on the presence of FRα-positive tumors identified using the VENTANA FOLR1 (FOLR1-2.1) RxDx Assay^[27]^. Subjects were randomly distributed (1:1) to either receive MIRV at a dose of 6 mg/kg (determined using adjusted ideal body weight) via intravenous infusion every three weeks or undergo a chemotherapy regimen, chosen by the study investigator until disease progression occurred or unacceptable side effects arose^[27]^. In total, 227 patients were designated to the MIRV group, while 226 were allocated to the chemotherapy group^[28]^. Among these, 425 participants received a minimum of one dose of their assigned therapy, with 218 in the MIRV group and 207 in the chemotherapy group, all of whom were included in the safety evaluation cohort^[28]^.

A complete response (CR) was characterized by the total elimination of all target lesions, with any pathological lymph nodes showing a reduction in short-axis measurement to below 10 mm. Conversely, a partial response (PR) was defined as a minimum 30% decrease in the aggregate diameters of target lesions when compared to baseline measurements^[29]^.In the safety evaluation cohort, the median duration of treatment was 4.98 months for the MIRV group, while it was 2.96 months for the chemotherapy group. Among the specific chemotherapy regimens, the median durations were 2.3 months for topotecan, 2.76 months for PLD, and 3.80 months for paclitaxel^[28]^.

The primary efficacy endpoints included PFS, OS, and overall response rate (ORR). The treatment showed a median OS of 16.5 months, in contrast to 12.7 months for chemotherapy, with a significant difference (hazard ratio for death, 0.67; *P* = 0.005). The median PFS was 5.6 months compared to 4.0 months (hazard ratio 0.65; *P* < 0.0001), and the ORR was 42% versus 16% (*P* < 0.0001)^[28]^. In the MIRV group, 12 participants (5.3%) experienced a CR, indicating that all target lesions completely disappeared. Meanwhile, a PR was observed in 84 participants (37.0%), reflecting at least a 30% reduction in the overall diameters of target lesions. In comparison, the chemotherapy group had no cases of complete response, with 36 participants (15.9%) showing partial responses.

Other secondary endpoints evaluated included the duration of response, response of cancer antigen 125 (CA-125), and safety profiles. The median duration of response for the 96 responders in the MIRV group was 6.77 months, while it was 4.47 months for the 36 responders in the chemotherapy group (hazard ratio, 0.62). Additionally, a higher percentage of participants in the MIRV group showed a CA-125 response compared to those in the chemotherapy group (58.0% versus 30.3%)^[28]^.

### Safety evaluation

**Figure 3.**
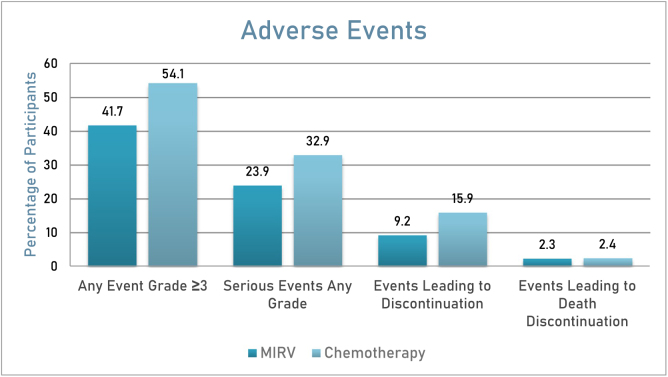
Adverse events observed in the MIRV group compared to the chemotherapy group.^[13]^

The clinical safety assessment involved 106 patients from Study 0417 (SORAYA Phase 3 Trial) who received a minimum of one dose of MIRV. Each patient was administered a single-agent dose of MIRV at 6 mg/kg (calibrated for ideal body weight) on the first day of every 3-week cycle (Fig. 3)^[30]^. The most commonly reported grade ≥3 adverse reactions (ARs) included visual impairment (7%) and keratopathy (9%). Permanent treatment discontinuation due to ARs was observed in 11% of patients, primarily linked to thrombocytopenia (2%) and intestinal obstruction (2%). Additionally, 20% of patients experienced dose reductions as a result of ARs, predominantly due to keratopathy (7%) and visual impairment (9%). Moreover, delays in dosage were required for 39% of patients, frequently related to keratopathy (11%) and visual impairment (15%)^[30]^.

The suggested dosage of MIRV is 6 mg/kg (based on adjusted ideal body weight), delivered as an intravenous infusion once every three weeks (21-day cycle) until either disease progression or intolerable toxicity arises. Unacceptable toxicities may include severe ocular issues such as visual impairment, keratopathy, dry eye, uveitis, photophobia, and eye pain^[15]^. Premedication with a corticosteroid, antihistamine, and antipyretic is recommended to mitigate potential adverse reactions. The prescribing guidelines feature a boxed warning about ocular toxicity, as well as additional cautions concerning pneumonitis, peripheral neuropathy, and embryo-fetal toxicity. Patients are instructed to use preventive ophthalmic topical steroids and lubricating eye drops to lower the likelihood of eye-related complications. An eye examination, which should include a visual acuity test and a slit lamp evaluation, is advised at baseline, every other cycle for the first eight cycles, and as clinically necessary^[31]^. Adjustments to the dosage should be determined by eye examination results, with immediate changes implemented for any new or worsening toxicities to maintain patient safety and treatment effectiveness.

MIRV represents a significant advancement over current standard treatments for PROC, including chemotherapy regimens such as paclitaxel, PLD, and topotecan. These traditional therapies often yield limited response rates and can be accompanied by substantial systemic toxicity, including myelosuppression, peripheral neuropathy, and gastrointestinal side effects. Treatment regimens may require frequent dose adjustments or changes due to these adverse effects, leading to challenges in maintaining effective therapy over time. In contrast, MIRV targets FRα-positive tumors, providing a more precise mechanism of action that may enhance therapeutic outcomes while reducing overall toxicity.

The traditional treatment landscape often lacks the specificity needed to address the diverse molecular characteristics of ovarian tumors, resulting in a one-size-fits-all approach. Moreover, the management of side effects in standard chemotherapy frequently necessitates additional interventions, such as the use of growth factor support for neutropenia. While MIRV does have specific ocular toxicities requiring careful monitoring and management, its potential for tailored therapy based on biomarker status offers a shift toward personalized medicine. This targeted strategy not only improves efficacy but also aims to preserve healthy tissue, making MIRV a valuable alternative in the evolving landscape of ovarian cancer treatment.

## Limitations

The majority of patients in the clinical trials have medium-to-high levels of folate receptor alpha (FRα) expression, which restricts the applicability of the findings to individuals with lower FRα levels. This limits the MIRV’s usefulness to a larger group of patients with ovarian cancer. The outcomes of some trials may not be generalizable if individuals have specific co-morbidities or have had particular previous treatments. Patients included in the trial may fare differently from those who have already received other experimental medicines. Many studies that focus on PFS and ORR as outcomes have relatively short follow-up periods. Our comprehension of the long-term advantages and hazards of treatment is limited by the incomplete evaluation of longer-term outcomes like OS and sustained quality of life.

The majority of the research focuses on patients with PROC, excluding people with platinum-sensitive ovarian cancer or patients with other ovarian cancer subtypes, such as mucinous or clear cell histologies, where MIRV may also be helpful but is still under investigation. A significant limitation is the short follow-up period of many studies, which may not adequately capture delayed adverse effects, including cumulative toxicities or treatment-related secondary malignancies. Another drawback is MIRV’s ocular toxicity, with patients commonly experiencing dry eyes, blurred vision, and keratopathy. While most ocular adverse effects are reversible, they can still negatively impact a patient’s quality of life, potentially requiring dose adjustments or discontinuation of treatment, reducing the drug’s overall effectiveness. Ocular toxicity management relies on dosage modifications and supportive care measures (e.g., eye drops), but the research may lack comprehensive, standardized protocols for preventing and addressing these adverse effects.

## Conclusion

The authorization of MIRV represents a significant advancement in treatment options for patients with FRα-positive primary ovarian, fallopian tube, or peritoneal cancer. This ADC employs a targeted therapeutic approach by concentrating on FRα found on cancer cells, thereby minimizing damage to healthy tissues and potentially overcoming drug resistance often encountered with standard chemotherapy. Clinical studies have underscored the encouraging effectiveness of this ADC, showing its ability to improve OS and PFS, as well as achieving a notable ORR.

However, the safety profile of MIRV necessitates vigilant monitoring, particularly concerning ocular toxicity, a significant adverse effect linked to the drug. With suitable dosage modifications and proactive management techniques, including premedication and routine ophthalmologic evaluations, the benefits of this therapy are expected to outweigh its risks. Looking forward, the outlook for MIRV in ovarian cancer treatment appears promising. Its approval offers a vital alternative for patients who have previously received systemic therapies, addressing a critical gap in cancer treatment. In summary, the emergence of targeted therapies such as MIRV signifies a meaningful progress in combating ovarian cancer, with the potential to enhance patient outcomes and improve quality of life globally.

## Data Availability

Not applicable.

## References

[1] Ali AT Al-Ani O Al-Ani F. Epidemiology and risk factors for ovarian cancer. Przegląd Menopauzalny = Menopause Rev 2023;22:93. 10.5114/pm.2023.128661 PMC10477765 37674925

[2] Sung H Ferlay J Siegel RL. Global cancer statistics 2020: GLOBOCAN estimates of incidence and mortality worldwide for 36 cancers in 185 countries. CA Cancer J Clin 2021;71:209–49. 33538338 10.3322/caac.21660

[3] cancer O. Ovarian cancer research alliance. Accessed September 22, 2024. https://ocrahope.org/get-the-facts/statistics/

[4] CDC. Ovarian cancer statistics. Ovarian Cancer. Published June 12, 2024. Accessed September 22, 2024. https://www.cdc.gov/ovarian-cancer/statistics/index.html

[5] Chen C Markossian TW Silva A. Epithelial ovarian cancer mortality among Hispanic women: sub-ethnic disparities and survival trend across time: an analysis of SEER 1992-2013. Cancer Epidemiol 2018;52:134–41. 29306788 10.1016/j.canep.2017.12.003

[6] Cancer of the ovary – cancer stat facts. SEER. Accessed September 22, 2024. https://seer.cancer.gov/statfacts/html/ovary.html

[7] Hu Y Taylor-Harding B Raz Y. Are epithelial ovarian cancers of the mesenchymal subtype actually intraperitoneal metastases to the ovary? Front Cell Dev Biol 2020;8:647. 32766252 10.3389/fcell.2020.00647 PMC7380132

[8] Hunter SM Anglesio MS Ryland GL. Molecular profiling of low grade serous ovarian tumours identifies novel candidate driver genes. Oncotarget 2015;6:37663–77. 26506417 10.18632/oncotarget.5438 PMC4741956

[9] Kuo KT Mao TL Jones S. Frequent activating mutations of PIK3CA in ovarian clear cell carcinoma. Am J Pathol 2009;174: 1597–601. 19349352 10.2353/ajpath.2009.081000 PMC2671248

[10] Djordjevic B Barkoh BA Luthra R. Relationship between PTEN, DNA mismatch repair, and tumor histotype in endometrial carcinoma: retained positive expression of PTEN preferentially identifies sporadic non-endometrioid carcinomas. Mod Pathol 2013;26:1401–12. 23599155 10.1038/modpathol.2013.67 PMC3720775

[11] Budiana ING Angelina M Pemayun TGA. Ovarian cancer: pathogenesis and current recommendations for prophylactic surgery. J Turkish Ger Gynecol Assoc 2019;20:47. 10.4274/jtgga.galenos.2018.2018.0119 PMC6501866 30362670

[12] Matulonis UA Sood AK Fallowfield L. Ovarian cancer. Nat Rev Dis Primers 2016;2:16061. 27558151 10.1038/nrdp.2016.61 PMC7290868

[13] Bosse K Rhiem K Wappenschmidt B. Screening for ovarian cancer by transvaginal ultrasound and serum CA125 measurement in women with a familial predisposition: a prospective cohort study. Gynecol Oncol 2006;103:1077–82. 16904167 10.1016/j.ygyno.2006.06.032

[14] Liu SY Ahsan Bilal M Zhu JH. Diagnostic value of serum human epididymis protein 4 in esophageal squamous cell carcinoma. World J Gastrointest Oncol 2020;12:1167–76. 33133384 10.4251/wjgo.v12.i10.1167 PMC7579729

[15] Powell CB Littell RD Landen CN. Cytological sampling of fallopian tubes using a hysteroscopic catheter: a multi-center study. Gynecol Oncol 2020;156:636–40. 31918994 10.1016/j.ygyno.2019.12.026

[16] Cortez AJ Tudrej P Kujawa KA. Advances in ovarian cancer therapy. Cancer Chemother Pharmacol 2018;81:17. 29249039 10.1007/s00280-017-3501-8 PMC5754410

[17] Galluzzi L Senovilla L Vitale I. Molecular mechanisms of cisplatin resistance. Oncogene 2012;31:1869–83. 21892204 10.1038/onc.2011.384

[18] Pilié PG Tang C Mills GB. State-of-the-art strategies for targeting the DNA damage response in cancer. Nat Rev Clin Oncol 2019;16: 81–104. 30356138 10.1038/s41571-018-0114-z PMC8327299

[19] Siddik ZH. Cisplatin: mode of cytotoxic action and molecular basis of resistance. Oncogene 2003;22:7265–79. 14576837 10.1038/sj.onc.1206933

[20] Food and Drug Administration (FDA). FDA approves mirvetuximab soravtansine-gynx for FRα positive, platinum-resistant epithelial ovarian, fallopian tube, or primary peritoneal cancer. Accessed June 3, 2024. https://www.fda.gov/drugs/resources-information-approved-drugs/fda-approves-mirvetuximab-soravtansine-gynx-fra-positive-platinum-resistant-epithelial-ovarian?utm_medium=email&utm_source=govdelivery

[21] Ab O Whiteman KR Bartle LM. IMGN853, a folate receptor-α (FRα)-targeting antibody-drug conjugate, exhibits potent targeted antitumor activity against FRα-expressing tumors. Mol Cancer Ther 2015;14:1605–13. 25904506 10.1158/1535-7163.MCT-14-1095

[22] Bogani G Coleman RL Vergote I. Mirvetuximab soravtansine-gynx: first antibody/antigen-drug conjugate (ADC) in advanced or recurrent ovarian cancer. Int J Gynecol Cancer 2024;34: 469–77. 38101816 10.1136/ijgc-2023-004924

[23] Matulonis UA Lorusso D Oaknin A. Efficacy and safety of mirvetuximab soravtansine in patients with platinum-resistant ovarian cancer with high folate receptor alpha expression: results from the SORAYA study. J Clin Oncol 2023;41:2436–45. 36716407 10.1200/JCO.22.01900 PMC10150846

[24] Moore KN Martin LP O’Malley DM. A review of mirvetuximab soravtansine in the treatment of platinum-resistant ovarian cancer. Future Oncol 2018;14:123–36. 29098867 10.2217/fon-2017-0379

[25] Dilawari A Shah M Ison G. FDA approval summary: mirvetuximab soravtansine-gynx for FRα-positive, platinum-resistant ovarian cancer. Clin Cancer Res 2023;29:3835–40. 37212825 10.1158/1078-0432.CCR-23-0991 PMC10592645

[26] O’Malley DM Matulonis UA Birrer MJ. Mirvetuximab soravtansine, a folate receptor alpha (FRα)-targeting antibody-drug conjugate (ADC), in combination with bevacizumab in patients (pts) with platinum-resistant ovarian cancer: final findings from the FORWARD II study. J Clin Oncol 2019;37:5520–5520.

[27] ClinicalTrials.gov. Study details | a study of mirvetuximab soravtansine vs. Investigator’s choice of chemotherapy in platinum-resistant, advanced high-grade epithelial ovarian, primary peritoneal, or fallopian tube cancers with high folate receptor-alpha expression. ClinicalTrials.gov ID NCT04209855. Accessed June 3, 2024. https://www.clinicaltrials.gov/study/NCT04209855

[28] Moore KN Angelergues A Konecny GE. Mirvetuximab Soravtansine in FRα-positive, platinum-resistant ovarian cancer. N Engl J Med 2023;389:2162–74. 38055253 10.1056/NEJMoa2309169

[29] Eisenhauer EA Therasse P Bogaerts J. New response evaluation criteria in solid tumours: revised RECIST guideline (version 1.1). Eur J Cancer 2009;45:228–47. 19097774 10.1016/j.ejca.2008.10.026

[30] ClinicalTrials.gov. Study details | a study of Mirvetuximab Soravtansine in platinum-resistant, advanced high-grade epithelial ovarian, primary peritoneal, or fallopian tube cancers with high folate receptor-alpha expression. ClinicalTrials.gov ID NCT04296890. Accessed June 3, 2024. https://clinicaltrials.gov/study/NCT04296890

[31] Fda C. Highlights of prescribing information. Accessed June 3, 2024. www.fda.gov/medwatch.

